# Therapy-induced senescence in breast cancer: an overview

**DOI:** 10.37349/etat.2024.00254

**Published:** 2024-07-25

**Authors:** Suraj Narayanan Chembukavu, Andrew J Lindsay

**Affiliations:** University of Bologna, Italy; Membrane Trafficking and Disease Laboratory, School of Biochemistry & Cell Biology, Biosciences Institute, University College Cork, Cork, T12 YT20, Ireland

**Keywords:** Drug resistance, therapy-induced senescence, membrane trafficking, iron metabolism, senotherapeutics

## Abstract

Outcomes for women with breast cancer have improved dramatically in recent decades. However, many patients present with intrinsic drug resistance and others are initially sensitive to anti-cancer drugs but acquire resistance during the course of their treatment, leading to recurrence and/or metastasis. Drug therapy-induced senescence (TIS) is a form of drug resistance characterised by the induction of cell cycle arrest and the emergence of a senescence-associated secretory phenotype (SASP) that can develop in response to chemo- and targeted- therapies. A wide range of anticancer interventions can lead to cell cycle arrest and SASP induction, by inducing genotoxic stress, hyperactivation of signalling pathways or oxidative stress. TIS can be anti-tumorigenic in the short-term, but pro-tumorigenic in the long-term by creating a pro-inflammatory and immunosuppressive microenvironment. Moreover, the SASP can promote angiogenesis and epithelial-mesenchymal transition in neighbouring cells. In this review, we will describe the characteristics of TIS in breast cancer and detail the changes in phenotype that accompany its induction. We also discuss strategies for targeting senescent cancer cells in order to prevent or delay tumour recurrence.

## Introduction

Breast cancer (BC) is the condition wherein some cells in the breast develop abnormally, leading to uncontrolled cell division, and ultimately the formation of tumours. It can be a devastating condition for millions of patients and their families worldwide [1]. Overall, BC is the most commonly occurring cancer and the most frequently diagnosed cancer in women [2]. Despite advances in detection and improved treatment strategies, about 30% of patients with early-stage BC have recurrent disease [3]. The cancer cells in these women have already spread to distant sites by the time they are diagnosed. While overall cancer survival rates have improved, the survival rates for patients with metastases have not [4]. A growing problem encountered in the clinic are tumours that have intrinsic or acquired resistance to targeted therapies. It is thus essential to understand the mechanisms of drug resistance and identify therapies that prevent or bypass resistance.

## Drug resistance in BC

The development of drug resistance accounts for up to 90% of BC deaths [3]. There are two predominant forms of resistance—intrinsic and acquired resistance, each accounting for 50% of patients with drug resistance [5].

Intrinsic resistance describes resistance that the patient possesses prior to exposure to drugs. This can be due to: (a) a pre-existing genetic mutation present in the tumour cells; (b) heterogeneity in tumours in which insensitive subclones are selected by drug treatment; or (c) activation of intrinsic pathways as defence mechanisms to certain drugs, such as drug efflux pumps, DNA damage repair (DDR) pathways and epigenetic modification.

Acquired resistance refers to the gradually declining efficacy of drug treatment. This can occur by: (a) activation of a second proto-oncogene that becomes the new driver gene; (b) mutation or altered expression levels of a drug target, rendering it insensitive to the drug; or (c) alterations of the tumour microenvironment (TME) [5].

### Drug efflux

In some cases, chemotherapy resistance can be caused by decreased intracellular drug accumulation due to dysregulated expression of ABC transporter genes. The ABC superfamily consists of 48 genes that can be divided into 7 subfamilies (*ABCA* to *ABCG*), among which *ABCB1*, *ABCC2*, and *ABCG2* have been reported to play a role in multi-drug resistance.

ABCB1 transporter contains two transmembrane domains that bind and hydrolyse ATP, following which multiple sites on the transporter bind and pump substrates such as etoposide, doxorubicin, paclitaxel, and vinblastine out of the cell [6–11]. Most tumour types, such as breast, liver, lung, kidney, colon, and rectal cancer express high levels of ABCB1 [12–14]. Unlike ABCB1, which pumps out amphipathic and lipid-soluble compounds, ABCC1 transporters exclusively pump organic anionic anti-cancer agents, such as epipodophyllotoxins, camptothecins, and methotrexate [15–18]. Intrinsic ABCC1 overexpression has been observed in BC, as well as prostate and lung cancer [15, 19, 20].

ABCG2 is largely expressed in BC but has also been reported in lung cancer and leukemia [21, 22]. It transports positively- and negatively- charged substrates such as mitoxantrone, bisantrene, and flavopiridols, as well as tyrosine kinase inhibitors (TKIs) such as gefitinib and imatinib [6, 21, 23].

### Alteration of drug targets

Targeted therapies (e.g., lapatinib, gefitinib, and erlotinib) act on specific target proteins to inhibit tumour development. Lapatinib is a dual epidermal growth factor receptor (EGFR)/HER2 inhibitor approved for use in HER2+ BC, while erlotinib and gefitinib target EGFR and are used to treat lung cancer. Cells can develop resistance to these TKIs through the alteration of drug targets; either via a secondary mutation, or by varying expression levels of the target protein [5]. This has been observed in non-small cell lung cancer (NSCLC) for EGFR targeting drugs, where 50% of patients treated with erlotinib and gefitinib develop a threonine-to-methionine mutation (T790M) within one year. This enhances the receptor tyrosine kinases (RTKs) binding affinity for ATP, and impairs drug binding [24–26]. Prolonged exposure to tamoxifen, a drug commonly used to treat hormone receptor-positive BC, has been reported to induce downregulation of its target estrogen receptor-alpha (ERα), resulting in resistance [27, 28].

### Enhanced DNA damage repair

Drugs like cisplatin and 5-fluorouracil (5-FU) cause cell death through the induction of DNA damage. However, genotoxic injury can upregulate DDR genes, such as FEN1, FANCG, and RAD23B leading to the initiation of senescence and development of insensitivity to chemotherapeutics [29, 30]. A suggested strategy to overcome this form of resistance is through the deregulation of DDR, though this might cause genomic instability and result in the initiation of another round of carcinogenesis [5].

### Epigenetic alterations

Epigenetic modifications such as DNA methylation, histone modification, and lncRNA regulation have been implicated in the induction of drug resistance. For example, tamoxifen and fulvestrant resistant MCF7 BC cells have been found to express diverse profiles of DNA methylation [31]. Fulvestrant resistance was characterised by ERα independent proliferation while tamoxifen resistance maintained ERα dependence but resulted in altered receptor-mediated gene regulation. In both cases, hypomethylation was more frequently observed than hypermethylation.

DNA methyltransferases (DNMT1, DNMT2, DNMT3A, DNMT3B, and DNMT3L) have been linked to tumour resistance. Particularly, overexpression of DNMT3A and DNMT3B was observed in tamoxifen resistant BC. It is suggested that radio- or chemo-sensitization of BC could be achieved through targeting these methyltransferases [32].

### Tumour heterogeneity and the microenvironment

Tumour heterogeneity can result in varying sensitivities to treatment and lead to the selection of cells with high tolerance to the drug [33–36]. There are four levels of heterogeneity: genetic, cell-type, metabolic, and temporal heterogeneity [37]. The presence of any form of heterogeneity makes it nearly impossible to kill all cells within a tumour with a single line of therapeutics. Heterogeneity can be tackled by combinatorial/cocktail therapies, such as FEC (5-FU, epirubicin and cyclophosphamide) for BC [5].

Another factor that can influence drug tolerance in cells is the TME. The TME includes extracellular matrix (ECM), immune cells, blood vessels, fibroblasts, and various other signalling molecules, and creates a permissive environment for cancer cells to survive and proliferate. Each TME factor can contribute to drug resistance through a number of strategies.

One such strategy is the manipulation of pH levels. Tumour cells can establish a “reverse pH gradient”, with increased intracellular pH and reduced extracellular pH level [38, 39]. Acidic intracellular pH has been reported to induce resistance to chemotherapeutics, by impairing the distribution of weak base anticancer drugs. This is known as “ion trapping”, and enables cancer cells to evade apoptosis [40, 41].

### Epithelial-mesenchymal transition

The process of epithelial cells detaching from each other and acquiring mesenchymal properties is known as epithelial-mesenchymal transition (EMT). It is essential for the initiation of metastasis [5]. The phenotypic changes associated with EMT can lead to drug resistance [42]. Transforming growth factor β (TGF-β) is an important cytokine required for the induction of EMT, and inhibition of its activity has been shown to reverse EMT, and increase sensitivity to drugs [43]. Multiple TGF-β inhibitors have been developed, but results in clinical trials have been disappointing to date [44]. Wnt and Hedgehog signalling pathways have also been reported to promote drug resistance through EMT. Wnt activates the Wnt/β-catenin pathway to promote an EMT-like phenotype upon treatment with trastuzumab (HER2-targeting therapeutic monoclonal antibody), while the Hedgehog pathway mediates resistance by promoting EMT upon treatment with anti-EGFR TKIs [45, 46].

## Senescence escape

Prolonged periods of targeted and chemotherapeutic treatment have been found to induce phenotypes resembling cellular senescence [47]. Cellular senescence is defined as a permanent or temporary cell cycle arrest. Senescent cells possess a number of hallmarks, including enhanced beta-galactosidase activity, senescence-associated secretory phenotype (SASP), cellular enlargement, and senescence associated heterochromatin foci (SAHF) [48–50]. SASP is characterized by a widespread change in protein expression in senescent cells, rendering it arrested in cell proliferation but still metabolically active.

Coppé et al. [51] have extensively characterized the SASP in human epithelial cells, and human and mouse fibroblasts. According to their work, the SASP can be classified into early events (alarm signals and mediators of tissue repair), and late events (anti-apoptotic proteins and inflammatory signals), which both negatively and positively regulate tumour progression, aging, and other pathologies. The wide impact of SASP can result in inhibition of cell division via genetic instability, oncogene inactivation, and promotion of immune clearance in cells [52].

**Figure 1:**
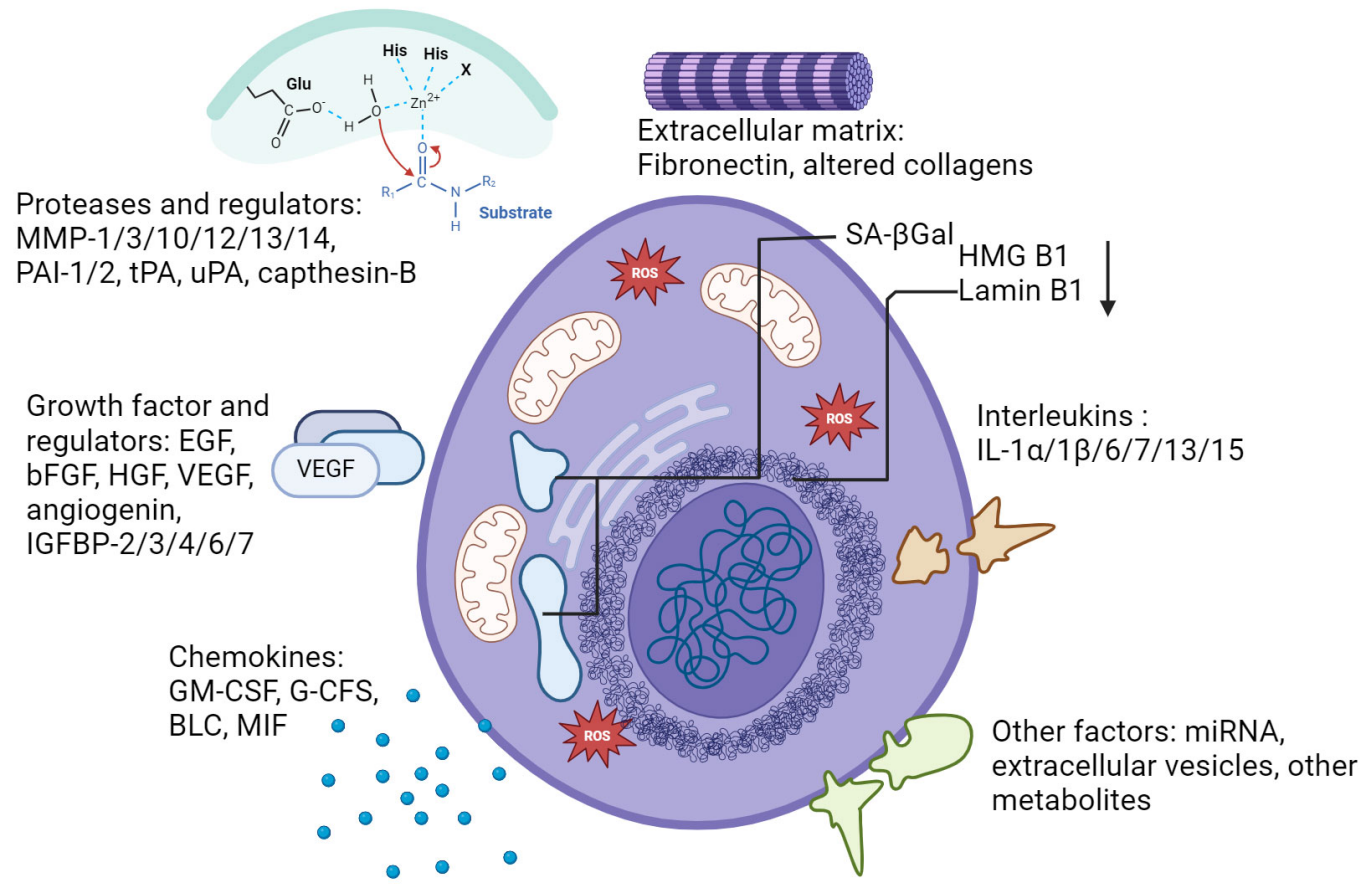
Characterization of senescence, senescence markers and SASPs. Characteristics of senescent cells include physiological and signalling markers such as growth factors, interleukins, chemokines, and proteases. Not all markers are conserved in every type of senescence. SASPs: senescence-associated secretory phenotypes. Created with BioRender.com

Senescent cells also differ in shape and size to a normal cell. They show increased protein content, an elevation in the amount of anti-apoptotic proteins, increased lysosomal hydrolase activity [presented through senescence-associated β-galactosidase (SA-βGal)], DNA damage, and telomere associated foci (TAFs) [53]. Some notable factors associated with SASP expression changes include pro-inflammatory interleukins (ILs; IL-6, IL-7, IL-1α, IL-1β, IL-13, IL-15), chemokines (GRO-α, GRO-β, MCP-1, MCP-2, eotaxin-3), growth factors (EGF, bFGF, HGF, VEGF), proteases (MMP-1, MMP-3, MMP-10, MMP-12, MMP-13, MMP-14, capthesin B), and ECM elements like fibronectin and altered collagen (Figure 1) [51, 53].

Senescence is increasingly recognized as an important mechanism of therapeutic resistance. In the following sections, we will provide an overview of the current understanding of the role of senescence in BC.

### Key senescence markers

In the previous section we discussed SASP, which is a crucial marker of senescence. However, the SASP is not conserved in every senescent cell, and there are common and unique features associated with different senescent phenotypes [54].

Oxidative stress related proteins have been proposed as potential markers of senescence, due to the multiple paths through which oxidative damage is incurred by senescent cells [55]. Upregulation of reactive oxygen species (ROS) acts as an inflammatory mediator for nuclear-factor kappa-light chain-enhancer of activated B cells (NF-κB) by upregulating the levels of inflammatory factor IL-1α, an upstream component of SASP [56]. Another hallmark of senescence is increased mitochondrial dysfunction, which is unsurprising as mitochondria are a major generator of ROS [57].

Alteration of lysosomal activity has been strongly linked to senescence, however, there are conflicting reports on whether lysosomal activity increases or decreases. There have been studies that suggest that senescence induces a decrease in lysosomal activity [58], while other studies report that pro-autophagic activity associated with senescence elevates lysosomal mass [59].

### Induction of senescence

Senescence can occur under a variety of circumstances. Cells naturally attain senescence after completing a finite number of divisions, at which point they reach the end of their replicative lifespan, called the Hayflick limit [60]. This is called replicative senescence and occurs due to the telomerase-induced reduction of telomere length after each replication cycle. Due to its intrinsic nature, it is also sometimes referred to as intrinsic senescence [61]. In contrast, extrinsic senescence is independent of telomerase activity, and can be categorized as onco-induced, tumour-suppressor loss-induced, or therapy-induced [61]. Onco-induced senescence (OIS) occurs upon oncogene activation, while tumour-suppressor loss-induced senescence (TSLIS) results from the inactivation of tumour suppressors, such as phosphatase and tensin homolog deletion on chromosome 10 (PTEN). Both OIS and TSLIS are reported to protect cells from neoplastic growth, and further transformation into malignancy [61].

Therapy-induced senescence (TIS) occurs as a result of drug therapy or radiation therapy [60]. Ionizing radiation (IR) is commonly employed in cancer therapy and has been shown to induce cellular senescence in doses ranging from 2 Gy to 10 Gy [62–64]. p21^Waf1/Cip1^ plays a critical role in cell cycle inhibition during early senescence, and p16^Ink4a^ was shown to maintain this phenotype [64, 65]. There is an upregulation in these cell cycle regulators upon the induction of TIS. This type of senescence has been reported to be telomere shortening independent, but ATM/Chk2, p38 MAPK, 5’-adenosine monophosphate-activated protein kinase (AMPK), NF-κB, and miR-34a dependent [66–68]. Radiation immediately activates the DNA damage response through the ATM/Chk2 pathway, followed by a series of slower events, including p38 MAPK activation, that contributes to SASP production [67]. The AMPK-NF-κB pathways become activated upon exposure to radiation and contribute to the induction of senescence by upregulating the expression of monocarboxylate transporter 1 (MCT1), which causes the export of lactic acid into the extracellular environment [68]. The inhibition of the AMPK-NF-κB pathway counteracts the acidification of the extracellular environment by reducing MCT1 levels.

There are other pathways and strategies that cells use to overcome IR-induced senescence. Expression of the multi-functional protein securin in IR-irradiated cells pushes them towards apoptosis and away from senescence [69]. Inhibition of glycolysis, or deficiency of PTEN also attenuates IR-induced senescence [68, 70].

A wide range of drugs (chemotherapies and targeted therapies) have been shown to induce senescence in tumour cells. *In vitro* and *in vivo* studies have reported that various drugs that induce TIS utilize a number of different pathways, including DNA damage, oxidative stress, post-translational modifications in proteolytic processing and kinase signalling [71]. To identify these senescence inducing drugs, Ewald et al. [72] screened a 4,160-compound library of known bioactive compounds and natural products and discovered over 225 cytotoxic compounds, 51 of which were potentially senescence inducing. Another drug screen identified inhibitors of the aurora kinase mitosis regulators as strong inducers of senescence [73].

In contrast to IR-induced senescence, drug TIS is reported to be independent of p53 status [74]. However, it was determined that each drug had its own unique mode of action. The most potent pro-senescence response was from DNA damaging agents like doxorubicin and cisplatin, while the weakest was from drugs targeting microtubules, such as taxol and vincristine [75].

Dosage is an important aspect of drug TIS and determine whether a cell becomes senescent or apoptotic; concentrations that are too low may not produce any effect while higher concentrations may induce apoptosis-mediated cell death [72, 76]. It was also noted that senescent markers were usually observed after a period of treatment ranging from 3 days to 7 days, and sometimes even longer [71].

A common feature of many senescence-inducing compounds is their ability to induce DNA damage via double- or single-stranded breaks [62, 77]. Although TIS does not demonstrate any telomere dependency, or telomeric shortening [78, 79], DNA damage in TIS usually occurs through an indirect alteration of DNA structure; for example, by inhibiting DNA methyltransferase using 5-azacytidine, or modifying chromatin structure using histone DNA acetyl inhibitors like sirtinol [80, 81].

Senescence can also be induced upon oxidative stress caused by chemotherapeutics such as pyrithione, which damage mitochondrial function [71]. This dysfunction can affect glycolysis, glutaminolysis, and other metabolic pathways that regulate double-strand break (DSB) repair and DNA damage checkpoints [82].

## TIS and iron metabolism

Mitochondrial dysfunction correlates strongly with cellular senescence and is accompanied by accumulation of intracellular iron (a nearly 30-fold increase in intracellular iron) [83, 84].

**Figure 2:**
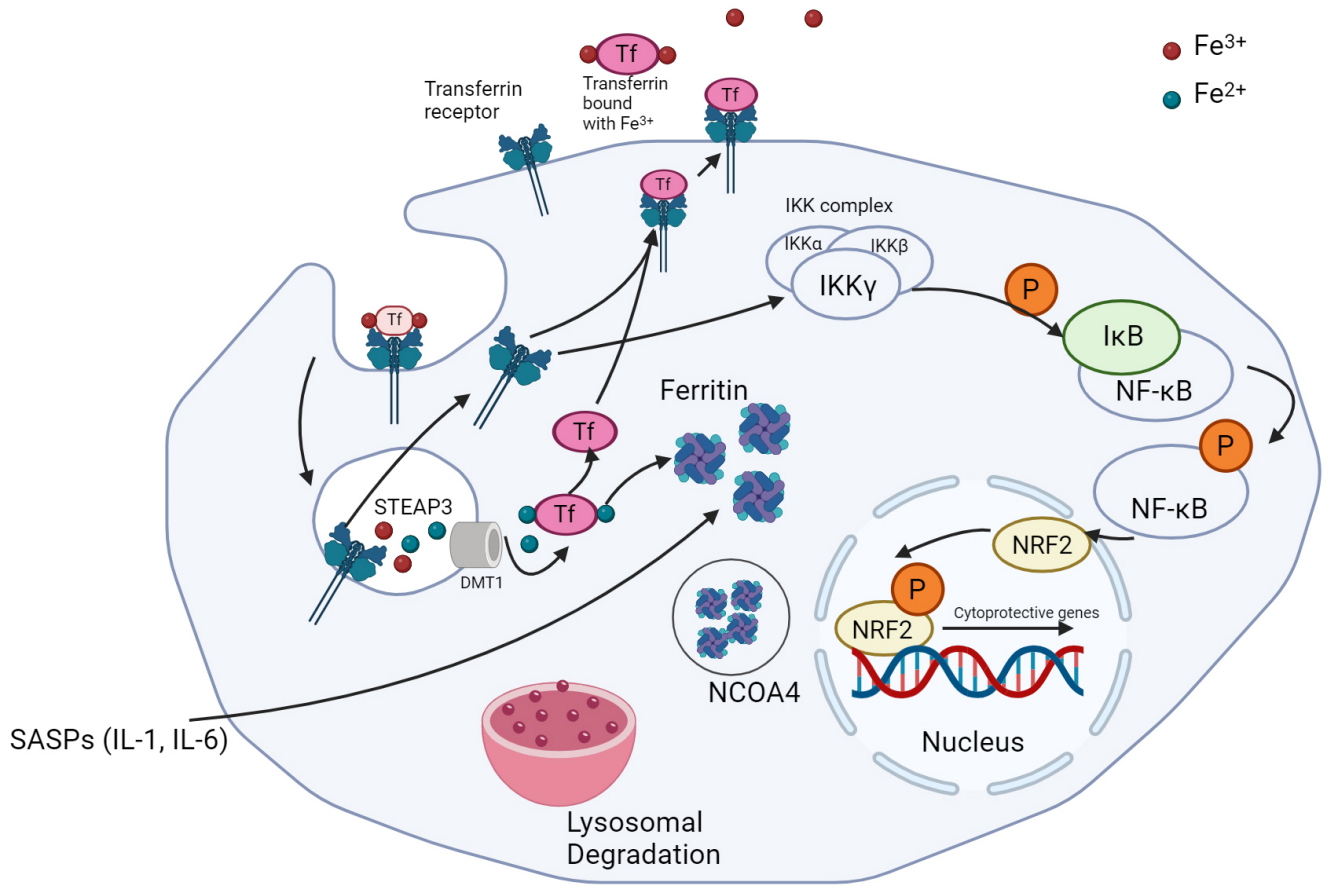
Iron metabolism in senescent cancer cells. Ferric iron is bound to transferrin and endocytosed with the help of TfR. Conversion of ferric to ferrous iron is followed by the binding of ferrous iron by ferritin. NCOA4 supports the degradation of these molecules via ferritinophagy. TfR is recycled to the plasma membrane and can also be utilized in the activation of the stress response via Nrf2 activation. TfR: transferrin receptor; Nrf2: nuclear factor-E2-related factor 2. Created with BioRender.com

Recently, Li et al. [84] reported a direct correlation between increased intracellular iron levels, mitochondrial dysfunction, and senescence. Intracellular iron assists in the generation of hydroxyl radicals through Fenton’s reaction. Fenton’s reaction involves the oxidation of ferrous iron to ferric iron in the presence of peroxide. It generates hydroxyl and hydroperoxyl radicals, which are unstable/reactive oxygen pollutants. With the critical role that iron plays in the generation of hydroxyl radicals, it is unsurprising that the induction of senescence impacts the expression levels of iron regulatory proteins like transferrin, transferrin receptor (TfR), and ferritin (Figure 2).

TfR is an integral membrane protein responsible for the uptake of iron and its expression is tightly controlled in normal cells. It transports iron-bound transferrin into the cell and is often overexpressed in BC to accommodate the increased iron demand for catalysis and biogenesis [85]. Elevated TfR levels have been observed in mouse embryonic fibroblasts induced to undergo IR-induced senescence [83, 86].

Neutrophil gelatinase-associated lipocalin (NGAL) is a secreted protein that can bind iron and plays a role in intracellular iron trafficking in mammals. It is correlated with tumour progression and is overexpressed in BCs with poor prognosis [87]. NGAL has been identified as a SASP factor in etoposide-induced senescence BC and mouse models of spinal cord injury [88, 89]. Furthermore, NGAL is upregulated in BC cells exposed to conditioned media from TIS and OIS cells [90].

Ferritin is a protein complex that captures intracellular ferrous iron (Fe^2+^) and converts it into ferric iron (Fe^3+^) through intrinsic ferroxidase activity, in order to reduce intracellular damage caused by ROS (Figure 2) [91]. FTH1 (ferritin-heavy chain) is the active component of this protein complex and directly correlates with ferritin activity [92]. FTH1 levels are higher in senescent cells and protect against ferroptosis, an iron-dependent cell death pathway [83].

Ferroportin is a transmembrane exporter responsible for the release of Fe^2+^ from the cell. It is downregulated in BC cells as a means to retain a larger amount of iron inside the tumour cells [86]. High levels of ferroportin have been associated with impaired tumour progression [93], and senescent cells display deregulated expression and altered subcellular localization [94]. Ferroportin seems to localise to intracellular compartments away from the plasma membrane, likely preventing it from partaking in iron efflux [83]. This impaired localization suggests an elevation in inactive transporters in senescent cells, which is consistent with senescence associated lysosomal degradation.

Another protein involved in the regulation of oxidative stress and ferroptosis is the nuclear factor-E2-related factor 2 (Nrf2). Nrf2 is a key transcription factor that regulates intracellular oxidative stress damage, and in turn, ferroptosis. It is a basic Leucine Zipper (bZIP) transcription factor that regulates the expression of proteins such as glutathione S-transferase, heme oxygenase 1, and NADPH quinone oxidoreductase [95]. Nrf2 activation is regulated by Kelch-like ECH-associate protein 1 (Keap1). Keap1 binds to non-phosphorylated Nrf2 in the cytoplasm, promoting its ubiquitination and proteasomal degradation [96]. NF-κB, when stimulated by inhibitor of NF-κB kinase (IKK), activates Nrf2 via phosphorylation, leading to the dissociation of Keap1 and its translocation into the nucleus [94, 97]. This protein complex then binds to apoptotic/electrophilic response elements, promoting transcription of cytoprotective genes [96, 98–101]. TfR can activate IKK and thus play a role in NF-κB signaling and activation of Nrf2 [102]. With senescent cells demonstrating a strong anti-apoptotic tendency despite the expression of apoptotic proteins, the levels of pNrf2 are a prominent indicator of senescent activity. Interestingly, silencing of Nrf2 has been associated with premature replicative senescence in human embryonic fibroblasts [80, 98].

In conclusion, TIS impacts iron metabolism in BC cells and affects iron transporters such as NGAL and TfR. It also affects the iron carriers such as ferroportin and ferritin, and the corresponding stress response pathways [103].

## Membrane trafficking pathways and senescence

A crucial element of membrane trafficking is the endosomal recycling pathway (ERP), which plays an important role in regulating the composition of the plasma membrane. This involves the internalization of cargo from the cell surface by a process called endocytosis. Endocytosed cargo is delivered to an intracellular organelle called the early endosome, where its fate is decided. It can either be returned to the cell surface via the ERP, or sent to lysosomes for degradation [104]. There are two main recycling pathways, based on the rate at which the process is carried out. In the fast recycling pathway, cargo is directly transported to the surface from the early endosome; in the slow recycling pathway the cargo is transported back to the plasma membrane via a perinuclear localized endosomal recycling compartment [105, 106].

A number of studies report that disruption of endocytosis results in the induction of cellular senescence [107–109]. For instance, the disruption of clathrin-mediated endocytosis (CME) caused by the destabilization of lysosomal membranes, results in DNA damage and changes in mitogenic signaling, leading to centrosome overduplication and induction of senescence [108].

There are a number of different mechanisms by which surface proteins can be internalized into the cell including CME, and caveolin-mediated endocytosis. Clathrin is a molecular scaffold that mediates the formation of vesicles at the plasma membrane [103], and caveolin is a cholesterol-binding protein that assists in the formation of cave-like structures in the plasma membrane, called caveolae [110].

### Caveolin-mediated endocytosis

Senescent cells have been reported to express high levels of caveolin-1 (Cav1), a major structural and functional unit of Caveolae-mediated endocytosis (CavME) [111], and CavME involves the fission of vesicles from the plasma membrane, followed by transport to endosomal compartments [112]. Cav1, which is the most common isoform of caveolin, is associated with a large number of signaling proteins, including RTKs, Src family kinase, and nitric oxide synthase 3 [113–115]. Deletion or overexpression of Cav1 results in altered signaling activity [116].

Cav1 also plays a role in lipid metabolism. Impairment of caveolin function, either due to mutation of the Cav1 gene or post-translational modifications of protein, leads to decreased levels of free cholesterol and increased neutral lipid storage in lipid droplets [117]. It has also been reported that Cav1 inhibits Nrf2 and other oxidative stress regulators, actively contributing to increasing cellular ROS [80, 118].

By virtue of its involvement in multiple signaling and metabolic pathways, Cav1 appears to play a central role in the development of senescence [119].

### Clathrin mediated endocytosis

Parallelly, another mechanism of endocytosis is CME, in which a group of adapter proteins are recruited to the plasma membrane to form clathrin-coated pits and mediate the invagination and fission of clathrin-coated vesicles [120]. Amphiphysin-1, a key regulator of CME is downregulated in senescent cells [121]. Notably, this downregulation coincided with reduced endocytosis of TfR [121]. Restoration of amphiphysin-1 restores the endocytic capacity of the cell. It is possible that downregulation of CME plays an important role in the induction of senescence [120].

The coatomer complex-1 (COPI) vesicle system transports cargo to and from the Golgi apparatus. The COPI transporters (encoded by COPB2, COPG1, and Arf1) regulate a number of membrane trafficking events, by mediating transport in the early secretory pathway [122]. COPI vesicle formation is regulated by the Arf family of GTPases. Beta PAK-interacting nucleotide exchange factor (βPIX), a GTPase activator, binds to G protein-coupled receptor kinase interacting proteins (GIT) and acts as an adaptor protein with Arf GAP activity (Arf GAP is a regulator of Arf family small GTPases, which are part of the COPI pathway) [123]. Studies have reported the βPIX-GIT complex plays a critical role in amphiphysin-1 cleavage [120]. βPIX levels reduce during the development of senescence, and silencing of βPIX or GIT both induce senescence [124]. Reduced βPIX levels result in a corresponding decrease in GIT levels, leading to destabilization of the paxillin-calpain complex, causing amphiphysin-1 cleavage and its deactivation [121]. Thus, senescence is directly linked to reduced CME via the downregulation of amphiphysin activity.

## Targeting TIS as an anticancer strategy

The differences in the morphology and functional properties of senescent cancer cells have led to many efforts to identify vulnerabilities that might be exploited to treat drug resistant cancers. One of the reported benefits of senescence in cancer is the restriction in tumour progression. It has also been reported that senescent properties can spread from one cancer cell to another [62, 125].

The detrimental effects of senescence induction via therapy include the promotion of EMT and concomitant increase in invasiveness of pre-malignant epithelial cells through the IL-6 and CXCL8-dependant pathways [126].

The SASP provides an opportunity for development of a “one-two punch” strategy in treatment of cancers with the use of senotherapeutics, therapeutic agents that target cellular senescence. Senomorphics (also known as senostatics) are a class of molecules that suppress some senescent properties and may even reverse the senescent phenotype. The field of senotherapeutic research is a growing area of drug discovery that encompasses targeted approaches, reverse-pharmacology, drug repurposing and other methodologies [127].

**Figure 3:**
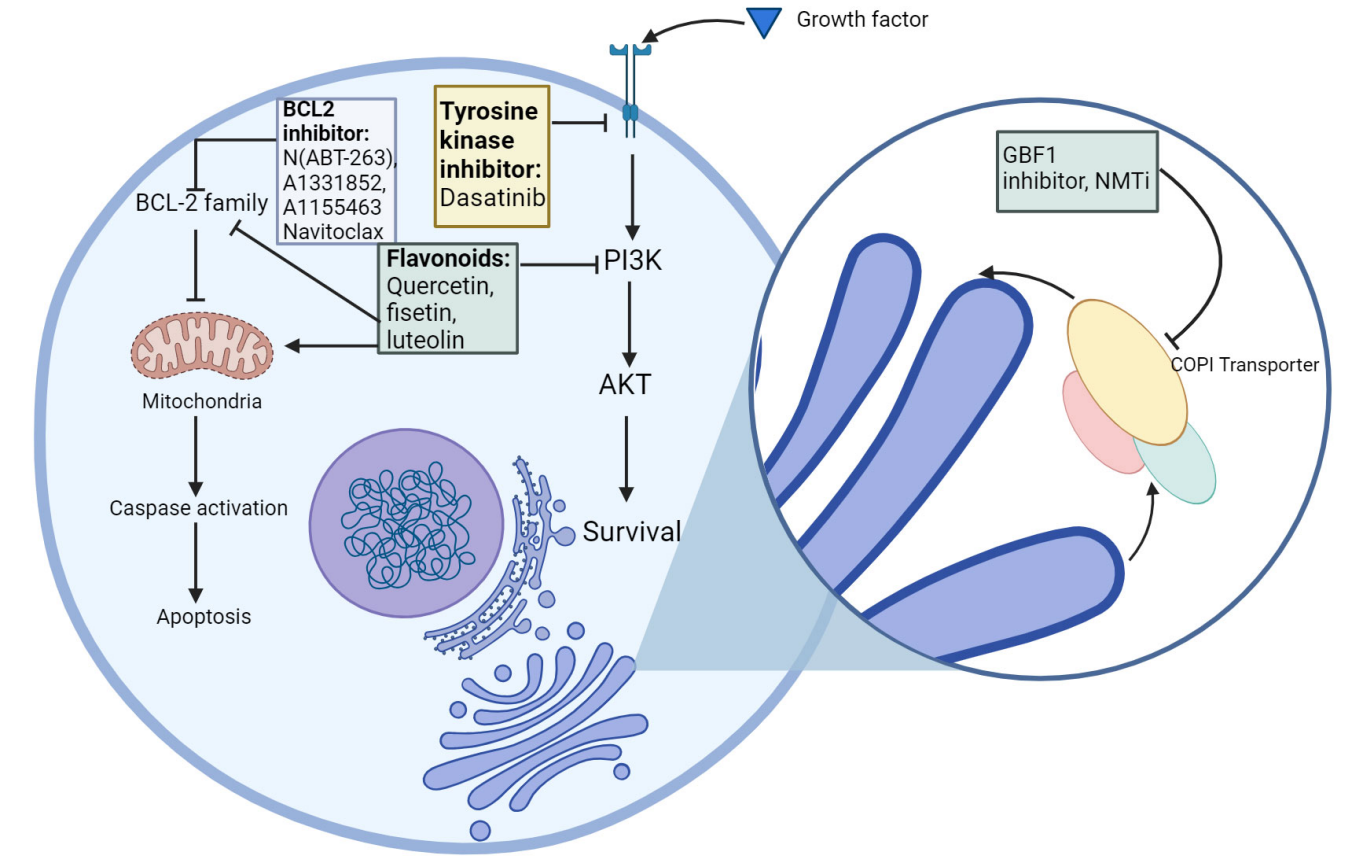
First generation senolytics and their corresponding target SCAPs. Currently approved senolytics, their molecular targets and their mode of action. The senolytics have been classified into their respective categories. SCAPs: senescent cell anti-apoptotic pathways. Created with BioRender.com

Senolytics target and kill senescent cells [53, 127] (Figure 3). Senomorphics suppress SASP, and in turn, decrease inflammation. The drawback of using senomorphics is that SASP is critical for wound healing [53].

It was first hypothesised that senescent cells upregulate anti-apoptotic and pro-survival pathways. This was confirmed by the continued survival of these cells upon upregulation of pro-apoptotic pathways, indicating the presence of factors that can counteract this cell death pathway. This led to the development of senolytics, which specifically target these anti-apoptotic pathways in senescent cells [128, 129]. Senescent cell anti-apoptotic pathways (SCAPs) possess similar properties to the anti-apoptotic pathways in B cell lymphoma and leukocytic leukaemia cells, as they release tissue destructive proapoptotic factors, but avoid undergoing apoptosis themselves [130, 131]. Senolytic compounds were considered more potent if they could target multiple SCAP nodes at the same time, as targeting single-node SCAPs like BCL2 pathway inhibitors [e.g., N(ABT-263), A1331852, A1155463] tended to affect a restricted range of senescent cells, and also increase risk of toxicity through off-target effects such as thrombocytopenia and neutropenia [130–132]. Targeting multiple SCAPs would allow them to attack a wider range of senescent cell types [133, 134].

Recent studies have identified the COPI pathway as a promising senolytic target in drug therapy-induced senescent cells [135]. Disrupting COPB2, a component of the COPI complex, results in Golgi dispersal and induction of apoptosis in senescent cells, indicating that this transport pathway may be a vulnerability in senescent cells [135]. The guanine-nucleotide exchange factor GBF1 is required for the activation of Arf GTPases. Conventional GBF1 inhibitors like brefeldin A selectively killed cells undergoing OIS, however, they have poor pharmacological properties and are not clinically approved. *N*-myristoylation inhibitors (NMTi), a clinically approved treatment for parasitic protozoan infections, were shown to phenocopy COPI inhibitors and act as potent senolytics [135, 136]. These work by preventing the addition of a myristoyl group to the amino terminus of Arf GTPases. Lack of this key lipid modification leads to reduction in the levels of Arfs 1, 3, 5 and 6 and a corresponding downregulation of the COPI pathway.

Ideal senolytics should target several SCAPs, be administered orally, and be pre-approved by the United States Food and Drug Administration (FDA) [137]. A wide-ranging list of senotherapeutics have been identified to date, such as dasatinib, quercetin, fisetin, luteolin, curcumin and navitoclax, and are in varying stages of clinical development [128, 133, 134, 138–140].

## Conclusions

This review explores the various facets of therapy resistance, and drug TIS stands out as a major drug resistance mechanism in BC. It involves the induction of irreversible cell cycle arrest and the development of SASP in cancer cells in response to chemotherapeutic agents and targeted therapies.

One of the key advantages of TIS is its potential to restrict tumour progression by halting the proliferation of cancer cells. However, in contrast to apoptosis, which results in cell death, senescent cells remain metabolically active and can influence the TME through the SASP. With both tumour suppressive and tumour promoting properties, senescence can be a double-edged sword in cancer treatment.

Challenges remain in effectively harnessing the therapeutic potential of TIS. Senescent cells can develop resistance to apoptosis, allowing them to persist within TMEs, and potentially contribute to disease relapse. Additionally, the SASP generated can have diverse impacts on tumour progression complicating therapeutic strategies.

Substantial effort is currently focussed on developing senotherapeutics which selectively target senescent cells, or modulate SASP. These include senolytics, which induce apoptosis in senescent cells, and senomorphics which suppress SASP. These represent a promising approach towards eliminating therapy-resistant cancer cells and enhancing treatment efficacy.

Several studies have explored the impact of senescence on iron metabolism, and the role that endocytosis of iron and iron transporters plays in this. These pathways could provide new targets for developing novel strategies to overcome drug TIS.

In conclusion, targeting drug TIS holds promise for overcoming drug resistance in BC. TIS has the potential to restrict tumour progression and enhance treatment efficacy through the induction of cell cycle arrest and modulating the TME. However, further research is required to better understand the impact of senescence on tumour progression and to optimize TIS-based therapeutic strategies while mitigating any potential adverse effects.

## Abbreviations

AMPK: 5’-adenosine monophosphate-activated protein kinase
BC: breast cancer
Cav1: caveolin-1
CME: clathrin-mediated endocytosis
COPI: coatomer complex-1
EGFR: epidermal growth factor receptor
EMT: epithelial-mesenchymal transition
ERα: estrogen receptor-alpha
ILs: interleukins
IR: ionizing radiation
Keap1: Kelch-like ECH-associate protein 1
NF-κB: nuclear-factor kappa-light chain-enhancer of activated B cells
NGAL: neutrophil gelatinase-associated lipocalin
Nrf2: nuclear factor-E2-related factor 2
OIS: onco-induced senescence
ROS: reactive oxygen species
SASP: senescence-associated secretory phenotype
SCAPs: senescent cell anti-apoptotic pathways
TfR: transferrin receptor
TIS: therapy-induced senescence
TKIs: tyrosine kinase inhibitors
TME: tumour microenvironment
βPIX: beta PAK-interacting nucleotide exchange factor

## Declarations

### Copyright

© The Author(s) 2024.
